# Interactions of pharmacologically active snake venom sPLA_2_ with different cell lines

**DOI:** 10.1080/13102818.2014.965014

**Published:** 2014-11-11

**Authors:** Jordan Doumanov, Kirilka Mladenova, Radoslav Aleksandrov, Georgi Danovski, Svetla Petrova

**Affiliations:** ^a^ Department of Biochemistry, Faculty of Biology, Sofia University ‘St. Kliment Ohridski’, Sofia, Bulgaria

**Keywords:** MDCK cells, RPE-1 cells, A549 cells, hBest1, vipoxin, sPLA_2_

## Abstract

Secreted Phospholipases A_2_ (sPLA_2_s) represent a large family of structurally related enzymes, which target different tissues and organs and induce numerous pharmacological effects based on their catalytic specificity – hydrolysis of the *sn*-2 ester bond of glycerophospholipids. The neurotoxin vipoxin, isolated from the venom of *Vipera ammodytes meriodionalis*, is a heterodimeric postsynaptic ionic complex composed of two protein subunits – a basic and toxic His48 sPLA_2_ enzyme and an acidic, enzymatically inactive and non-toxic component. In this paper, for the first time, we demonstrate that vipoxin sPLA_2_ enzyme affects cell integrity and viability of four cell types and causes different cell responses. The most dramatic local tissue effects were observed with RPE-1 (retinal pigment epithelial) cells followed by A549 (adenocarcinomic human alveolar epithelial) cells and MDCK (Madin-Darby Canine Kidney epithelial) cells. Products of the enzymatic reaction, lysophospholipids and unsaturated free fatty acids, act as lipid mediators that can induce membrane damaging or can stimulate cell proliferation. Our preliminary results on the cytotoxic effect of vipoxin sPLA_2_ on A549 cells are promising in searching of its eventual anticancer potential.

## Introduction

Secreted phospholipases A_2_ (sPLA_2_s, EC 3.1.1.4) represent a superfamily of Ca^2+^dependent esterases that catalyse the hydrolysis of the *sn-*2 ester bond in 1,2-diacyl-3-sn-phosphoglycerides releasing free fatty acids and lysophospholipids. They play important roles in key biochemical processes such as generation of pro-inflammatory lipid mediators (prostaglandins and leukotrienes), remodelling of phospholipid membranes and regulation of lipid metabolism. Snake venom sPLA_2_ enzymes (GI and GII groups) are responsible for major local tissue damage during envenoming often manifested by myonecrosis, lymphatic vessel damage and inflammatory response provoking different ways of tissue behaviour.[1] Significant progress has been made in understanding the pathogenesis of snake venom-induced local tissue damage, but it is unbelievable how limited is still our knowledge about its mechanism. The extensive studies on the role of sPLA_2_ in cell death have postulated that (1) at high toxin concentrations, cells die of necrosis (cell and organelle swelling, ATP (Adenosine triphosphate) depletion, increased plasma membrane permeability, release of macromolecules and inflammation); (2) at lower toxin concentrations they undergo apoptosis (ATP-dependent process, characterized by cell shrinkage, chromatin condensation, plasma membrane blebbing and caspase activation); and (3) at still lower PLA_2_ levels, cells proliferate (synthesize inflammatory mediators).[2,3]

We investigated the interactions of the sPLA_2_ subunit of vipoxin with different cell lines in order to analyse (1) the local tissue damage potential of toxic snake venom sPLA_2_; and (2) their effect on cell membrane integrity, cell viability and response. We have already demonstrated that sPLA_2_ catalytic activity is directly related to its neurotoxic, haemolytic and anticoagulant effects.[4] The used cell lines (MDCK, MDCK-hBest1 (MDCK cells, stably transfected with the gene encoding human Best1 (hBest1)protein [5])), RPE-1 and A549, were selected as natural pharmacological targets of sPLA_2_.

## Materials and methods

### Phospholipase A_2_ toxin

The neurotoxin vipoxin was isolated from the crude venom (collected from various specimens) of *Vipera ammodytes meridionalis* (Thracian Herpetological Society and National Centre of Infectious and Parasitic Diseases, Bulgaria) using ion-exchange chromatography on SP-Sephadex C-50 (Pharmacia, Sweden) according to the procedure described previously.[6] The separation of vipoxin's subunits was modified and optimized as described.[7] Active fractions were used immediately after dialysis or lyophilized and stored at −20 °C (253.15 K). The protein homogeneity after each purification step was assessed by SDS-PAGE.[8] Protein concentration was measured by the method of Smith et al. [9].

### Substrates and chemicals

4-Nitro-3-(octanoyloxy)benzoic acid (NOBA) is from Enzo Life Sciences, Inc. (USA); p-bromophenacyl-bromide (pBPB), 8-anilinonaphthalene-1-sulphonic acid (ANS), Dulbecco's modification of Eagle's medium (DMEM), foetal calf serum (FCS), penicillin, streptomycin, G418 disulphate salt, MTT ([3-(4, 5-dimethyl-2-thiazolyl)-2,5-diphenyl-2H-tetrazolium bromide]) reagent and Trypan blue are from Sigma-Aldrich Co. (St. Louis, MO, USA). All other chemicals and solvents used were of analytical grade.

### Enzyme activity assay

Phospholipase A2 activity was assayed using synthetic substrate NOBA as described by Cho and Kezdy [10], Holzer and Mackessy [11] and modified for 96-well plates by Ponce-Soto et al. [12]. The standard assay mixture contained 2.25 × 10^−4^ L of buffer (1 × 10^−2^ mol L^−1^ Tris-HCl, 5 × 10^−3^ mol L^−1^ CaCl_2_, 1 × 10^−1^ mol L^−1^ NaCl, pH 8.0), 1.5 × 10^−5^ L of NOBA (1.5 × 10^−3^ mol L^−1^ in acetonitrile) and 1 × 10^−5^ L of appropriately diluted sPLA_2_ (about 1 × 10^−6^ mol L^−1^) in a final volume of 2.5 × 10^−4^ L. After the addition of sPLA_2_, the mixture was incubated for up to 40 minutes at 37 °C (310.15 K). The absorbance at 4.25 × 10^−7^m (425 nm) was read in five-minute intervals. The enzyme activity (expressed as the initial velocity of the reaction) was calculated based on the increase in absorbance after 15 minutes. All assays were conducted in triplicate using Dynex microplate reader (Dynex Technologies, USA). Enzymatically non-active sPLA_2_ was prepared by specific chemical modification of His48 residue (catalytic site group) with pBPB, which completely inhibited the enzyme activity.[13] We used ANS as a fluorescent ‘hydrophobic probe’ in order to visualize microscopically the interaction of cells with sPLA_2_ (Nikon Eclipse TS 100 microscope). The enzyme labelled with ANS retained completely its catalytic activity against NOBA.[14]

### Cell cultures

A549, MDCK II, Best1-transfected MDCK II and RPE-1 cells were grown in DMEM supplemented with 10% FCS, streptomycin (1 × 10^−7^ kg L^−1^), penicillin (6 × 10^−8^ kg L^−1^) and G418 (5 × 10^−4^ kg L^−1^ only for the transfected MDCK II) at 37 °C (310.15 K) and humidified air containing 5% CO_2_.

### In vitro cytotoxicity testing of sPLA_2_

For MTT test,[15] MDCK II and MDCK II-Best1 cells were plated at initial concentration of 5 × 10^4^ cells per well, A549 and PRE-1 were plated at initial concentration of 6 × 10^4^ cells per well.

After 24 h of incubation at 37 °C (310.15 K), the cells (at a density of 5 × 10^4^ cells per well) were incubated for two hours with different concentrations of sPLA_2_ (from 0.5 to 2.5 × 10^−6^ mol/L^−1^). The cells not treated with sPLA_2_ served as a control and were used to normalize the viability data. After certain time intervals, MTT solution was added to each well at a final concentration of 0.5 × 10^−3^ kg L^−1^ and the plates were incubated at 37 °C (310.15 K) for four hours. The MTT formazan product was dissolved by addition of 1.1 × 10^−4^ L acidified 2-propanol (in 4 × 10^−2^ mol L^−1^ (0.04 N) HCl) to each well. The absorbance was detected at 5.4 × 10^−7^ m (540 nm) using Dynex microplate reader (Dynex Technologies, USA). Cell survival rate was calculated as ratio of the (absorbance of the treated wells)/(absorbance of the control wells) × 100%.

To establish the kinetics of interaction sPLA_2_‑cells and appearing of eventual morphological changes, cells were incubated with sPLA_2_ (1.5 × 10^−6^ mol L^−1^) for different times, visualized with an inverted microscope (XDS-2) and stained for death cells with 0.5% Trypan blue.

## Results and discussion

Snake venoms are some of the most complex multifunctional mixtures of pharmacologically active proteins and polypeptides interfering in various physiological systems. Among them sPLA_2_ enzymes turn out to be the most toxic, which can provoke diverse pharmacological effects – neurotoxicity, myotoxicity, cardiotoxicity, anticoagulant effects, hemolytic activity, haemorrhage, organ or tissue damage.[1] However, at the same time, they have extremely important biochemical role in many physiological processes such as maintenance of membrane homeostasis, membrane repair, cell proliferation, inflammation, signal transduction, etc.

Here, for the first time, we investigated the biological effect of separated and purified sPLA_2_ subunit of the neurotoxin vipoxin, isolated from *Vipera ammodytes meridionalis*, on different cell lines – MDCK II, MDCK II-Best1, A549 and RPE-1. These preliminary studies clearly demonstrated the role of the enzymatic activity and its specificity on membrane integrity, cell viability and proliferation. We used snake venom sPLA_2_, not only to establish its toxicity on different tissue cell membranes, but also to demonstrate its important function in cell signalling pathways and its functional relationship to human secreted PLA_2_ GIIA that is responsible for many physiological processes.

**Figure 1.**
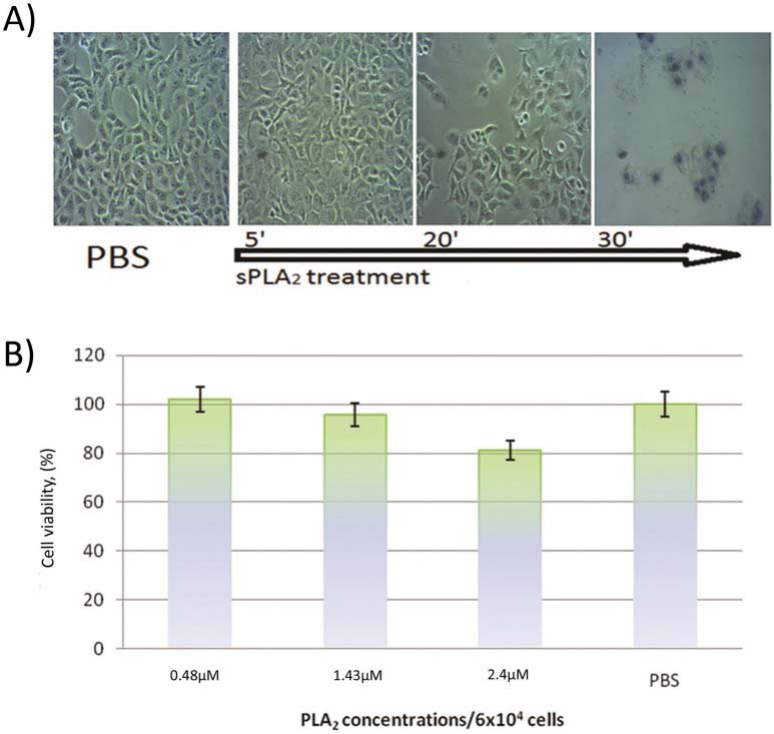
(A) Kinetics of MDCK II cells treatment with sPLA_2_ (1.5 × 10^−6^ mol L^−1^ and Trypan blue staining at 30 min). (B) *In vitro* cytotoxic effect of pure sPLA_2_ (0.5 to 1.5 × 10^−6^ mol L^−1^ on MDCK II cells after two hours of exposure to different snake venom sPLA_2_ concentrations. Cell viability is determined by MTT assay (the MTT value of control sample exposed only to PBS buffer is defined as 100% viability). Data from the experiments performed in triplicate are expressed as mean ± SD.

**Figure 2.**
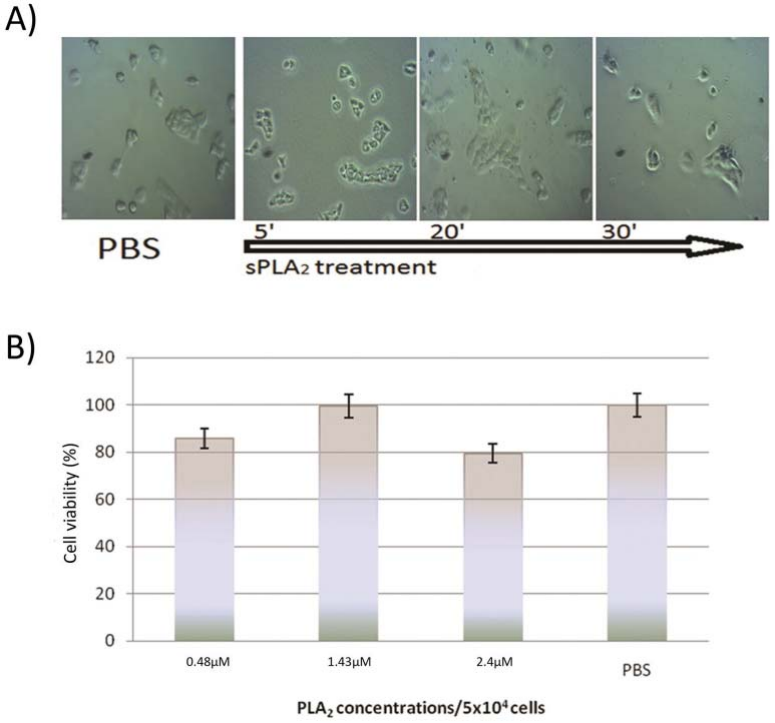
(A) Kinetics of Best-transfected MDCK II cells treatment with PLA_2_ (1.5 × 10^−6^ mol L^−1^ and Trypan blue staining at 30 min). (B) *In vitro* cytotoxic effect of pure sPLA_2_ (0.5 to 1.5 × 10^−6^ mol L^−1^ on MDCK II-Best1 cells after two hours exposure to different snake venom sPLA_2_ concentrations.

**Figure 3.**
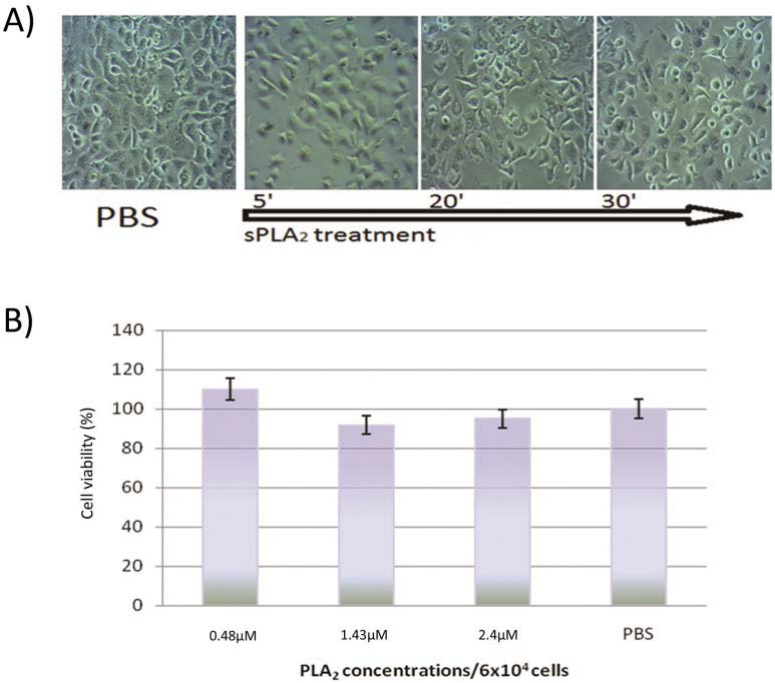
(A) Kinetics of A549 cells treatment with PLA_2_ (1.5 × 10^−6^ mol L^− 1^ and Trypan blue staining at 30 min). (B) *In vitro* cytotoxic effect of pure sPLA_2_ (0.5 to 1.5 × 10^−6^ mol L^−1^ on A549 cells after two hours of exposure to different snake venom sPLA_2_ concentrations.

**Figure 4.**
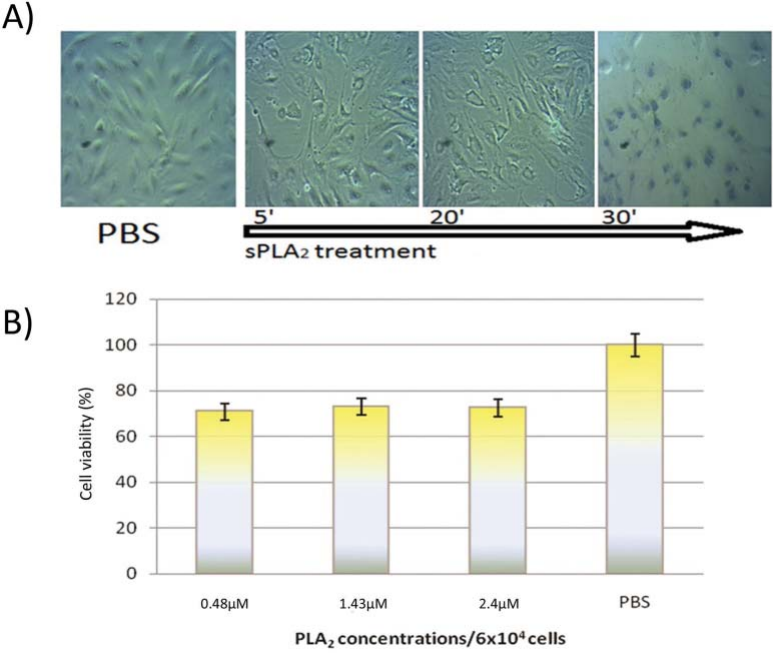
(A) Kinetics of RPE-1 cells treatment with PLA_2_ (1.5 × 10^−6^ mol L^−1^ and Trypan blue staining at 30 min). (B) *In vitro* cytotoxic effect of pure sPLA_2_ (0.5 to 1.5 × 10^−6^ mol L^−1^ on RPE-1 cells after two hours of exposure to different snake venom sPLA_2_ concentrations.

Cells from four different lines were exposed to sPLA_2_ at a concentration of 0.5 to 2.5 × 10^−6^ mol L^−1^ (1 to 5 kg^−9^) per 5 × 10^4^ cells for two hours. At different time intervals light microscopic images were taken for monitoring the morphological changes. The results are shown in Figures 1(A)–4(A). The *in vitro* cytotoxicity test (Figures 1(B)–4(B)) was carried out two hours after the cell treatment with different sPLA_2_ concentrations.

We established similar behaviour and degree of reduction in viability of MDCK II, MDCK II-Best1 and A549 cells in time- and concentration-dependent manner but observed different morphological changes during the course of incubation with sPLA_2_ – many swollen, burst and death cells, as well as membrane remnants.

Best1 transfected MDCK II cells showed delayed response to sPLA_2_ treatment (Figure 2(B)). At concentration of 1.5 × 10^−6^ mol L^−1^, sPLA_2_ did not affect viability of MDCK II-Best1 cells and they demonstrated higher membrane stability, most likely due to the expressed transmembrane Best1 protein (calcium-dependent chloride channel), the composition, physicochemical and structural properties of phospholipid bilayers. At higher sPLA_2_ (2.5 × 10^−6^ mol L^−1^) concentration, the viability was reduced by 20% and we observed morphological changes similar to those of MDCK II cell line.

Under the conditions of the experiment, pure sPLA_2_ caused negligible cytotoxic effect on A549 cell line and at lower enzyme concentrations cell proliferative process or activation of mitochondrial enzymes was established (Figure 3(B)). After two hours of exposure to 2.5 × 10^−6^ mol L^−1^ sPLA_2_, only about 10% decrease in MTT values was detected, suggesting slight effect on mitochondrial functions. On the contrary, light microscopic images clearly showed typical necrotic morphological changes as swelling, disintegration, blebbing and increased Trypan blue staining at 30 minutes of incubation.

The most striking effect of sPLA_2_ catalytic and pharmacological activities was observed in the case of RPE-1 cells. Immediately after the cells exposure to the toxin, we monitored necrotic morphological changes (swelling, membrane disruption and protrusions) as well as cell death at 30 min of incubation revealed by Trypan blue staining (Figure 4(A)). MTT test values showed also higher sensitiveness of RPE-1 cells to snake sPLA_2_ – at lowest used sPLA_2_ concentration (0.5 × 10^−6^ mol L^−1^), cell viability was reduced by 30% (Figure 4(B)). It was unexpectedly that higher sPLA_2_ concentrations did not decrease and even slightly increase the MTT values, demonstrating a different mechanism of interaction of sPLA_2_ with RPE-1 cells as well as a possibility for accumulation of lysophospholipids and unsaturated fatty acids (as products of the reaction which are destructive by themselves) to act as signal molecules and stimulate mitochondrial enzymes and/or proliferation in adjacent cells.

**Figure 5.**
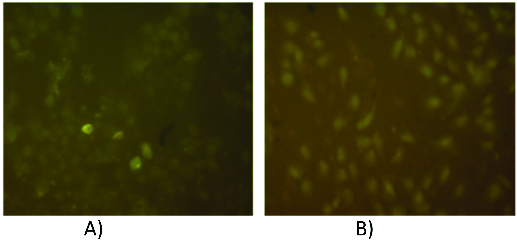
Fluorescence micrographs of MDCK II (A) and RPE-1 (B) cells after incubation with 1.5 × 10^−6^ mol L^−1^ ANS-labelled sPLA_2_.

Chemically modified, with pBPB, pure sPLA_2_ had no effect on cell viability and membrane integrity of all treated cell lines for the whole exposure period. This fact definitely shows that the mechanism of cytotoxicity is dependent on sPLA_2_ enzymatic activity. Fluorescently labelled with ANS sPLA_2_ adsorbs on the cell membranes of MDCK and RPE-1 cells with different affinity as shown in Figure 5.

Recently, Samel et al. [16] reported on the adverse effects of secreted PLA_2_s from *Viperalebetina*, *Viperaberusberus* and *Najanajaoxiana* venom on different target cells (platelets, cancer cell types and bacteria). Closely related to the investigated vipoxin's sPLA_2_, *Viperaberusberus* sPLA_2_ inhibited significantly (in concentration of 7.2 × 10^−6^ mol L^−1^) the viability of leukaemia lineK-562 cells and caused apoptotic cell death. Ammodytoxin A (AtxA), a monomeric snake venom sPLA_2_ from *Vipera ammodytes ammodytes*, was established rapidly to internalize into motoneuronal NSC34 cells, inducing characteristic neurotoxic sPLA_2_ cell damage and apoptosis.[17] The authors suggest specific binding to the motoneuronal cell surface, followed by internalization and enzymatic activity-dependent induction of apoptosis and extensive extra- and intra-cellular free fatty acid release, necessary to induce cell death. We could propose similar mechanism of adsorption and internalization for sPLA_2_ from *Vipera amodytes meridionalis* as fluorescently labelled enzyme was visualized inside the cells. Furthermore, the cell contact and enzyme adsorption on the outer membrane leaflet are essential for interfacial catalysis.

Depending on the membrane composition and structure of the cells, enzyme activity of sPLA_2_ caused morphological and functional changes characteristic for cell damage (direct effect) but longer exposure of the cells to sPLA_2_ could demonstrate the indirect effect of the liberated reaction products that induce cell death. It will be necessary to throw a light on the thin border between the two possible enzyme dependent mechanisms for cell response – apoptosis and necrosis.

## Conclusion

Toxic sPLA_2_ subunit of vipoxin, isolated from *Vipera ammodytes meridionalis* snake venom, affects cell membrane integrity of different cell lines, leading to increased permeability, membrane disruption and blebbing. At prolonged exposure to the enzymatic action of sPLA_2_, cells swell, burst and die. Their cell viability is dependent on sPLA_2_ concentration and its interfacial binding surface, phospholipid specificity, membrane composition, organization and properties. The most sensitive to toxic sPLA_2_ were RPE-1 cells. Results indicated that the effects caused by vipoxin's sPLA_2_ subunit should be studied in the light of the induction of both apoptosis and necrosis.
